# Hospital Festivals and Meetings

**Published:** 1895-03-30

**Authors:** 


### HOSPITAL FESTIVALS AND MEETINGS.

### ROYAL LONDON OPHTHALMIC HOSPITAL, MOORFIELDS.

The annual meeting of the governors of this hospital was held on Tuesday. Mr. Charles Gordon, in the absence of the President (Sir John Lubbock), occupied the chair, and there were also present the Rev. Prebendary Whittington, the Rev. J. W. Pratt, Messrs. Bedell, John Tweedy, H. P. Sturges, J. Lea Smith, and C. Ridley Smith. The annual report, of which the Chairman moved the adoption, showed that during the year there were 1,981 in-patients, as against 1,973 in the previous year. The total number of out-patients was 24,511, the attendances of out-patients reaching the large number of 113,555. In 1,863 cases where spectacles had been ordered, either a gift of spectacles had been made, or material assistance had been given towards their purchase. The income for the year had been £7,627; the ordinary expenditure £6,983, and the extraordinary—the greater part of it in connection with the new site—£4,094. A new site has been acquired for the hospital in the City Road, near Old Street, at a cost of £2,854. Plans are being considered, and it is hoped that the laying of the foundation-stone will take place at an early date. The report concluded with an expression of deep regret at the death of Mr. Whitaker Hulke, president of the College of Surgeons, who for many years had been a surgeon of the hospital. Mr. John Tweedy seconded. The report adopted, votes of thanks were passed to Sir John Lubbock the president, Mr. John Deacon the treasurer, the committee of management, and the medical staff. The editors of metropolitan journals were thanked for the publicity which they had given to the claims of the hospital. The rule under which the election of honorary medical and surgical officers is vested in a committee composed of members of the committee of management and of the medical board, and all personal canvassing is forbidden, was re-enacted. On the motion of Mr. Bedell, a vote of condolence with the widow and family of the late Mr. Whitaker Hulke was passed. The proceedings closed with a vote of thanks to the chairman.

### UNIVERSITY COLLEGE HOSPITAL.

On Friday, March 22nd, the annual general meeting was held in the board-room of the hospital, the chair being taken by Mr. Henry Lucas, who is also chairman of the committee. Commenting upon the report, Mr. Lucas pointed out that there had been a considerable falling off in the people's contribution fund during the past year, the total amount being only £360. This was to be accounted for by commercial depression, and also because the renewing of collecting boxes had been an additional expense. A general scheme was now under consideration for the gradual rebuilding of a new hospital of not less than 300 beds, and though nothing approaching the full sum required for such a purpose had as yet been obtained by the committee, there were sufficient funds in hand for the building of a new block in the rear of the present hospital which it was hoped would much improve the accommodation. The committee confidently looked forward to the commencement of this new wing during the present year, and they appealed earnestly for donations and subscriptions. Those already received or promised amounted to rather over £3,000. Annual subscriptions, donations, &c., had increased, but the committee had to call attention to the very serious debt of some £12,000, which had increased by £1,000 since last year. The festival dinner had been a great success, and they had also to record a munificent donation of £2,000 from Mrs. Nathaniel Montefiore for the endowment of a bed in memory of her late father, Sir Isaac Lyon Goldsmid, Bart. The adoption of the report was seconded by Mr. J. W. Bangs and agreed to. The re-election of the committee was then proceeded with, and with the usual votes of thanks the meeting closed.

### POPLAR HOSPITAL FOR ACCIDENTS.

There is not much variety, as a rule, in the proceedings at hospital annual meetings, and little or no individuality about their conduct in nine cases out of ten; but this cannot be said of the meeting of Governors of the Poplar Hospital for Accidents, which took place on Tuesday last. It was a friendly gathering together of those genuinely interested in, and fond of, the institution they helped to support. A large number of working-men governors were present, and the spirit of cheery co-operation which obviously animated all there was an object-lesson indeed for those who looked below the surface, and realised how this almost unique condition has been brought about at Poplar. The Hon. Sydney Holland (chairman), Mr. Corner, Colonel Feneran (house-governor), and others were on the platform.

The chair was taken by Mr. Coles, vice-president, who, in moving the adoption of the report, said he thought there was cause for much gratification at the results of past work, as shown in the figures contained therein. The smaller sums and the workmen's items were proofs of much loving-kindness. He could not pass over the resignations of Mr. Corner and Mr. Brownfield, two of the hospital's oldest friends, without expressing a sense of the deep gratitude owed to them for life-long labour. Mr. Coles made one or two friendly criticisms upon the accounts, paid a tribute, which he said he knew to be quite out of order, to the indomitable energy of Mr. Holland, the chairman, and concluded with the hope that the yearly festival dinner in June might be well attended, and that every one would bring as many friends as possible.

Mr. Holland said he had an explanation to make with regard to one item in the accounts, that of rates. Hitherto the guardians, seeing what the hospital was doing, and realising how much of their work was done by it, had not required any payment of rates, but now the London County Council insisted upon their being rated, the amount being £61. The rating of hospitals was a serious question which would have to be considered by Government. With regard to funds, £10,000 had been collected during the past year, £2,000 more than ever before, and the collection had only cost the hospital 5 per cent. Lord Cadogan had stated somewhere the other day that no hospital could hope to succeed unless it were permanently in debt, but it was their intention to keep free from debt or curtail expenses. The new hospital had cost £31,000, every penny of which had been paid. By way of illustration of the urgent need for the existence of the Poplar Hospital, Mr. Holland read out to the meeting the House-Surgeon's record of one day's work, the Monday in the present week—80 cases of accidents were brought in, the first at a-quarter to nine a.m., the last at ten minutes to one p.m., and that was by no means an unusual number. In addition, there were 130 out-patients seen. He wished to thank the press for encouragement given, and the working men for their hearty support, they having contributed £650 during the past year, on one day collecting in the streets over two hundredweight of coppers. They had also to thank Mr. C. W. Cayzer for his generous gift of £500, and Sir Donald Smith, who had actually given them £1,500. A great want in the hospital was a mortuary chapel, and their friend Mr. Speyer, knowing this, had offered to provide it—a welcome gift indeed. Mr. Holland spoke with warm appreciation of the work of the House Surgeon (Mr. Radford), of the Matron (Miss Vacher), and her nurses, incidentally remarking that it was felt that the latter were most inadequately paid, the salaries being from £20 to £25 a year, and this was a question he felt sure the governors would wish to be looked into. In conclusion, he cordially invited everyone to go over the wards of the hospital before leaving the building.

Other speeches followed, one and all showing how happy is the feeling which exists between those who work for and interest themselves in the Poplar Hospital. Perhaps the most valuable testimony came from the workmen themselves, several of whom spoke, and the burden of whose remarks was the pride taken in "their" hospital, and the gratitude universally felt towards the surgical and nursing staff for unwearying kindness to all who came under their care. Miss Vacher, who was present with some of the nurses, must have valued the spontaneous tribute from one of the men, who "did not believe there was another matron in London like our matron," and that "nothing said by us could be sufficiently high on account of the nurses and the staff."

Messrs. Corner, Brownfield, and Bowkett, the retiring members of the staff, were unanimously elected honorary consulting surgeons, with many expressions of gratitude and goodwill, and it was announced that Mr. P. W. Howse, F.R.C.S., and Mr. T. H. Openshaw, F.R.C.S., would fill two of the vacant posts.

The meeting closed with a vote of thanks to Mr. Coles for presiding, and most of those present afterwards paid a visit to the wards.

